# Prevalence of FRAX risk factors and the osteoporosis treatment gap among women ≥ 70 years of age in routine primary care across 8 countries in Europe

**DOI:** 10.1007/s11657-021-01048-8

**Published:** 2022-01-22

**Authors:** Eugene McCloskey, Jeetandera Rathi, Stephane Heijmans, Mark Blagden, Bernard Cortet, Edward Czerwinski, Peyman Hadji, Juraj Payer, Kerry Palmer, Robert Stad, James O’Kelly, Socrates Papapoulos

**Affiliations:** 1 Centre for Metabolic Bone Diseases, University of Sheffield, Sheffield, UK; 2 Carrig Medical Centre, Cork, Ireland; 3 ResearchLink, Linkebeek, Belgium; 4 Ashgate Medical Practice, Chesterfield, UK; 5 Department of Rheumatology and EA 4490, University-Hospital of Lille, Lille, France; 6 Department of Bone and Joint Diseases, FHS, Jagiellonian University Medical College, Kopernika 32, 31-501 Krakow, Poland; 7 Frankfurt Center of Bone Health, Frankfurt, Germany; 8 Philipps-University of Marburg, Marburg, Germany; 9 Faculty of Medicine, 5th Department of Internal Medicine in University Hospital Bratislava, Comenius University, Bratislava, Slovakia; 10 Amgen Ltd, Uxbridge, UK; 11 Amgen Europe GmbH, Rotkreuz, Switzerland; 12 Leiden University Medical Center, Leiden, Netherlands

**Keywords:** Fragility fracture, Fracture Risk Assessment Tool (FRAX), Risk factors, Observational study, Osteoporosis, Treatment gap

## Abstract

***Summary*:**

We studied whether elderly women at risk for fractures receive primary care treatment to prevent fracture. We found that across Europe, women at risk are often not identified, and less than half of such women receive appropriate treatment. Finally, women diagnosed with osteoporosis are much more likely to receive treatment.

**Purpose:**

To examine the relationship between risk factors for fragility fracture (FF) and osteoporosis (OP) treatment gap in elderly women across Europe, and compare the prevalence of risk factors between countries.

**Methods:**

Demographic and clinical information was collected from women ≥ 70 years visiting primary care physicians in Belgium, France, Germany, Ireland, Poland, Slovakia, Switzerland, and the UK. Increased risk of FF was defined by the presence of 1 or more criteria (history of fracture, 10-year fracture probability, or *T*-score ≤  − 2.5).

**Results:**

There were 3798 women in total. Treatment gap (proportion at increased risk of FF not receiving treatment for OP) varied from 53.1 to 90.8% across countries, and the proportion of patients at increased risk of FF varied from 41.2 to 76.1%. Across countries, less than 50% of patients with increased risk of FF had a diagnosis of OP. Previous fracture was the most common risk factor, with similar prevalence across most countries; other risk factors varied widely. The treatment gap was reduced in patients with an OP diagnosis in all countries, but this reduction varied from 36.5 to 79.4%. The countries with the lowest rates of bone densitometry scans (Poland, France, and Germany; 8.3–12.3%) also had the highest treatment gap (82.2 to 90.8%).

**Conclusions:**

This study highlights differences across Europe in clinical risk factors for fracture, rates of densitometry scanning, and the rates of OP diagnosis. More emphasis is needed on risk assessment to improve the identification and treatment of elderly women at risk for fracture.

**Supplementary Information:**

The online version contains supplementary material available at 10.1007/s11657-021-01048-8.

## Introduction

Osteoporosis (OP) is a systemic disease characterized by low bone mass and deterioration of bone tissue, which results in bone fragility and an increased risk of fracture [1]. Bone loss accelerates in the year just before menopause and remains high as long as 4–8 years after menopause, with women losing on average 10% of their bone mineral density (BMD) during the menopausal transition [2]. In 2015, the prevalence of BMD-defined OP in women aged 50 or more across the EU5 (France, Germany, Italy, Spain, and UK) and Sweden ranged from 21.8% in the UK to 23.1% in Italy [3]. The related fractures were found to impose a substantial economic burden in these countries, with direct costs in 2017 ranging from € 1199 million in Sweden to € 8176 million in Germany [3]*.*

Dual-energy X-ray absorptiometry (DXA) is the most widely used technique for measuring BMD, upon which the diagnosis of OP is generally based. Tools such as the FRAX fracture risk assessment tool developed at the University of Sheffield, which integrates clinical risk factors and BMD, are being commonly used to calculate the risk of fracture [4]. Increasingly, FRAX values are used to set national or international thresholds for assessment and/or treatment to identify patients to be targeted for pharmacotherapy [5–8]. Despite this, only a minority of women eligible for treatment receive appropriate treatment [9]. In 2010, the estimated treatment gap in Europe varied from 26% in Ireland to 78% in Poland [10, 11]. More recent studies have indicated that the treatment gap persists; e.g., > 75% of respondents (representative of the general French population) 50 years or older who reported a fracture had not been diagnosed with OP, or received appropriate care [12], and the treatment gap in women in the EU5 and Sweden was estimated as 73% for 2017 [3]. The primary analysis from our recent study of 8 European countries confirmed this large treatment gap (74.6%) in women aged ≥ 70 years at increased risk of FF in routine primary care across Europe [13]. The gap appeared to be related to a low rate of OP diagnosis. In this analysis, we wished to examine the relationship between fracture risk factors and the treatment gap across the 8 countries. In addition, the study provided an opportunity to compare the prevalence of fracture risk factors over the same countries.

## Methods

Detailed methods for this cross-sectional, multicenter, observational study have been published previously [13].

### Study design, data collection method, study population, and recruitment

Following informed consent, community-dwelling women 70 years or older across 8 countries (Belgium, France, Germany, Ireland, Poland, Slovakia, Switzerland, and the UK) filled a short questionnaire when spontaneously visiting their GP between 28 March 2018 and 26 October 2018. Demographics, baseline characteristics, and other information (reason for consultation, any known OP diagnosis, risk factors for fracture, comorbidities, and OP medications used in the last 10 years) were collected using the questionnaires and patient medical records.

### Primary and key secondary objectives

This publication reports further analysis from the study (the primary analysis of which has been published; see [13]). The objective of this analysis was to compare the prevalence of clinical risk factors for fracture in the 8 countries in the study, and examine any relationship between fracture risk factors and the treatment gap across these countries.

### Definition of increased risk of FF

A patient was considered at increased risk of FF if at least 1 of the following 3 criteria was met: (a) history of fracture after the age of 50 (hip, spine, wrist, or other OP-related fractures); (b) increased 10-year probability of both hip *and* major osteoporotic fracture (calculated using the FRAX tool without BMD) above the country-specific threshold, which was calculated using an approach similar to that of the National Osteoporosis Guidance Group, UK ([13, 14]; see Online Resource Table S1); (c) BMD *T*-score ≤  − 2.5 for lumbar spine, total hip, or femoral neck.

### Statistical methods

No formal hypothesis was tested. A sample size of 4000 (500/country) was expected to estimate the primary outcome for each participating country with sufficient precision. All analyses were descriptive. Baseline patient characteristics, OP medication use, and clinical risk factors were summarized by country. The primary outcome (OP treatment gap) was summarized by country for important clinical risk factors and for OP diagnosis (yes or no).

## Results

A total of 3798 patients were enrolled; approximately 500 per country (except for Switzerland, n= 205), across 153 sites.

### Baseline characteristics

The median age of patients ranged from 76.0 in Ireland, Poland, and Slovakia to 79.0 in France; median body mass index (BMI) ranged from 25.5 kg/m^2^ in Switzerland to 28.7 kg/m^2^ in Slovakia. The most common reason for consultation was “follow-up to known disease” in 7 of 8 countries (ranging from 36.4% in Poland to 73.8% in Slovakia); in France, “medication refill” (48.8%) was the most common reason (Table 1).

**Table 1:** Baseline characteristics, OP diagnosis, comorbidities, clinical risk factors, and 10-year risk of FF by country

	**Belgium (*N* = 505)**	**France (*N* = 543)**	**Germany (*N* = 506)**	**Ireland (*N* = 500)**	**Poland (*N* = 505)**	**Slovakia (*N* = 534)**	**Switzerland (*N* = 205)**	**UK (*N* = 500)**	**Overall *N* = 3798**
Age, median (Q1, Q3), years	78.0 (74.0, 82.0)	79.0 (74.0, 84.0)	78.0 (74.0, 81.0)	76.0 (73.0, 81.0)	76.0 (72.0, 80.0)	76.0 (72.0, 80.0)	78.0 (74.0, 83.0)	77.0 (73.0, 82.0)	77.0 (73.0, 82.0)
BMI, median (Q1, Q3), kg/m^2^	27.1 (23.8, 30.2)	25.6 (22.8, 29.7)	26.5 (23.2, 29.7)	27.1 (23.9, 30.5)	27.5 (25.2, 31.2)^*^	28.7 (25.7, 32.1)	25.5 (22.8, 29.1)	26.6 (23.8, 30.7)	26.9 (23.9, 30.5)^**^
Reason for consultation, *n* (%)									
Follow-up to known disease	226 (44.8)	168 (30.9)	368 (72.7)	209 (41.8)	184 (36.4)	394 (73.8)	141 (68.8)	288 (57.6)	1978 (52.1)
Medication refill	151 (29.9)	265 (48.8)	57 (11.3)	39 (7.8)	165 (32.7)	68 (12.7)	21 (10.2)	16 (3.2)	782 (20.6)
New symptoms/complaints	110 (21.8)	72 (13.3)	72 (14.2)	170 (34.0)	134 (26.5)	55 (10.3)	41 (20.0)	170 (34.0)	824 (21.7)
Other	18 (3.6)	38 (7.0)	9 (1.8)	82 (16.4)	22 (4.4)	17 (3.2)	2 (1.0)	26 (5.2)	214 (5.6)
Known OP diagnosis, n (%)	125 (24.8)	107 (19.7)	82 (16.2)	129 (25.8)	76 (15.0)	144 (27.0)	62 (30.2)	79 (15.8)	804 (21.2)
DXA assessment	188 (37.2)	62 (11.4)	62 (12.3)	223 (44.6)	42 (8.3)	139 (26.0)	91 (44.4)	137 (27.4)	944 (24.9)
Increased risk of FF—*n* (%)	286 (56.6)	376 (69.3)	295 (58.3)	241 (48.2)	259 (51.3)	220 (41.2)	156 (76.1)	244 (48.8)	2077 (54.7)
With a diagnosis of OP^a^	107 (37.4)	88 (23.4)	60 (20.3)	101 (41.9)	62 (23.9)	95 (43.2)	62 (39.7)	66 (27.0)	641 (30.9)
At least one comorbidity, *n* (%)	438 (86.7)	471 (86.7)	438 (86.6)	416 (83.2)	479 (94.9)	504 (94.4)	183 (89.3)	432 (86.4)	3361 (88.5)
RA	18 (3.6)	27 (5.0)	25 (4.9)	16 (3.2)	16 (3.2)	21 (3.9)	7 (3.4)	21 (4.2)	151 (4.0)
Diabetes	104 (20.6)	91 (16.8)	187 (37.0)	69 (13.8)	148 (29.3)	214 (40.1)	44 (21.5)	96 (19.2)	953 (25.1)
Hypertension	372 (73.7)	368 (67.8)	401 (79.2)	331 (66.2)	435 (86.1)	487 (91.2)	137 (66.8)	309 (61.8)	2840 (74.8)
Osteoarthritis	242 (47.9)	348 (64.1)	117 (23.1)	237 (47.4)	235 (46.5)	129 (24.2)	129 (62.9)	265 (53.0)	1702 (44.8)
COPD	40 (7.9)	39 (7.2)	53 (10.5)	58 (11.6)	32 (6.3)	35 (6.6)	13 (6.3)	57 (11.4)	327 (8.6)
Clinical risk factors for FF—*n* (%)									
Previous fracture	159 (31.5)	148 (27.3)	151 (29.8)	155 (31.0)	156 (30.9)	178 (33.3)	96 (46.8)	157 (31.4)	1200 (31.6)
Hip	22 (4.4)	20 (3.7)	17 (3.4)	21 (4.2)	16 (3.2)	19 (3.6)	8 (3.9)	22 (4.4)	145 (3.8)
Spine	26 (5.1)	21 (3.9)	28 (5.5)	14 (2.8)	22 (4.4)	24 (4.5)	31 (15.1)	12 (2.4)	178 (4.7)
Wrist	62 (12.3)	54 (9.9)	53 (10.5)	52 (10.4)	69 (13.7)	73 (13.7)	22 (10.7)	50 (10.0)	435 (11.5)
Other (except skull, finger and toe fractures)	77 (15.2)	70 (12.9)	97 (19.2)	86 (17.2)	74 (14.7)	89 (16.7)	51 (24.9)	90 (18.0)	634 (16.7)
Parental hip fracture	53 (10.5)	75 (13.8)	42 (8.3)	35 (7.0)	41 (8.1)	44 (8.2)	27 (13.2)	49 (9.8)	366 (9.6)
Current smoker	34 (6.7)	27 (5.0)	35 (6.9)	33 (6.6)	28 (5.5)	25 (4.7)	14 (6.8)	35 (7.0)	231 (6.1)
Glucocorticoid use	18 (3.6)	17 (3.1)	23 (4.5)	33 (6.6)	21 (4.2)	9 (1.7)	14 (6.8)	41 (8.2)	176 (4.6)
Alcohol (≥ 3 units per day)	6 (1.2)	3 (0.6)	4 (0.8)	9 (1.8)	0 (0.0)	2 (0.4)	7 (3.4)	19 (3.8)	50 (1.3)
Femoral neck T-score – median (Q1, Q3) [n]	-1.9 (-2.4, -1.1) [124]	-1.4 (-2.0, -0.4) [35]	-1.3 (-2.3, -0.8) [48]	-1.5 (-2.1, -0.7) [203]	-2.0 (-2.7, -1.4) [21]	-1.4 (-2.0, -0.9) [124]	-2.0 (-2.6, -1.2) [86]	-1.3 (-2.1, -0.4) [86]	-1.6 (-2.3, -0.8) [727]
10-year fracture probability without BMD – median (Q1, Q3), %									
Hip fracture	8.8 (4.8, 13.9)	8.8 (4.7, 13.8)	7.6 (4.9, 11.7)	7.7 (5.1, 12.3)	3.7 (2.3, 6.5)^*^	6.1 (3.9, 9.3)	12.2 (7.4, 20.1)	7.2 (4.1, 12.1)	7.2 (4.1, 11.9)^**^
Major OP fracture	18.3 (13.1, 24.3)	18.3 (11.2, 25.5)	16.6 (12.1, 22.7)	18.0 (13.8, 25.0)	9.5 (6.7, 13.8)^*^	14.5 (10.9, 20.1)	29.3 (23.7, 39.3)	18.3 (13.0, 24.6)	16.6 (11.5, 23.9)^**^

*BMD*, bone mineral density; *BMI*, body mass index; *COPD*,, chronic obstructive pulmonary disease; *DXA*, dual-energy X-ray absorptiometry; *FF*, fragility fracture; *OP*, osteoporosis; *RA*, rheumatoid arthritis

*N*, number of patients enrolled in full analysis set. Percentages based on number of patients enrolled in full analysis set

^a^Percentages in this row are based on *N* at increased risk of FF in each country (given in the row above)

^*^N = 485. ^**^N = 3778

**Note:** Patients may be counted in more than one comorbidity

Across countries, most patients did not have an OP diagnosis (Table 1); the proportion with an OP diagnosis ranged from 15.0% in Poland to 30.2% in Switzerland. Belgium, Switzerland, and Ireland had a relatively high proportion of patients with DXA assessments (37.2 to 44.6%); other countries ranged from 8.3% (Poland) to 27.4% (UK). For interpretation of BMD and *T*-score data, it should be noted that DXA assessments were available only for a subgroup of 944 patients (24.9% of the study cohort).

France and Switzerland had the highest proportion of patients at increased risk of FF (69.3% and 76.1%), and Slovakia had the lowest (41.2%). Across countries, < 50% of the patients with increased risk of FF had a diagnosis of OP, ranging from 20.3% in Germany to 43.2% in Slovakia (Table 1).

Baseline characteristics and reasons for consultations for patients at increased risk of FF were similar to the total populations in all countries (Online Resource Table S2).

### Comorbidities

Most patients in all countries reported at least one comorbidity, ranging from 83.2% in Ireland to 94.9% in Poland (Table 1). Of the 5 comorbidities on which data were collected, hypertension was the most prevalent (ranging from 61.8% in the UK to 91.2% in Slovakia), and rheumatoid arthritis (RA) the least prevalent (ranging from 3.2% in Ireland and Poland to 5.0% in France). The countries with the highest proportion of patients at increased risk of FF also had the highest prevalence of osteoarthritis (France 64.1% and Switzerland 62.9%), and the country with the lowest proportion of patients at increased risk of FF had the lowest prevalence of osteoarthritis (Slovakia 24.2%). However, in general, there was no obvious pattern between the prevalence of comorbidities and the fracture risk between countries.

### Clinical risk factors for FF

In the study population as a whole, previous fracture was the most commonly reported clinical risk factor (Table 1), with similar prevalence across most countries (from 27.3% in France to 33.3% in Slovakia); except for Switzerland, which had a relatively high prevalence (46.8%) as a result of more frequent spine (15.1%) and “other” (24.9%) fractures. Variation was seen across countries for the risk factors of parental hip fracture (7.0% in Ireland to 13.8% in France), current smoking (4.7% in Slovakia to 7.0% in the UK), glucocorticoid use (1.7% in Slovakia to 8.2% in the UK), and alcohol intake of 3 or more units daily (0.0% in Poland to 3.8% in the UK). Median femoral neck *T*-score ranged from -1.3 in Germany and the UK to -2.0 in Poland and Switzerland.

Previous fracture was also the most common risk factor in patients with increased risk of FF (Online Resource Table S2). As expected, prevalence was higher than for the total patient group, ranging from 39.4% in France to 80.9% in Slovakia. Variation across countries was seen in clinical risk factors including parental hip fracture (12.0% in Ireland to 17.3% in France and Switzerland), current smoking (4.5% in Slovakia to 8.3% in Switzerland), glucocorticoid use (3.2% in Slovakia to 12.9% in Ireland), RA (3.8% in Switzerland to 8.1% in Germany), alcohol intake of 3 or more units daily (0.0% in Poland to 4.1% in the UK), and median femoral neck *T*-score (-1.3 in Germany to -2.5 in Poland).

### 10-year probability of hip and major OP fracture

Ten-year probability of hip and major OP fracture in patients without BMD assessment was similar across most countries (Table 1); hip: 6.1% in Slovakia to 8.8% in Belgium and France; major OP: 14.5% in Slovakia to 18.3% in Belgium, France, and the UK. Values were notably lower for Poland (hip: 3.7%; major OP: 9.5%) and higher for Switzerland (hip: 12.2%; major OP: 29.3%). A similar pattern was seen for patients at increased risk of FF (Online Resource Table S2); as expected, values were higher than for all patients (hip: 6.4% in Poland to 14.5% in Switzerland; major OP: 13.7% in Poland to 32.8% in Switzerland).

### OP treatment gap

**Fig. 1:**
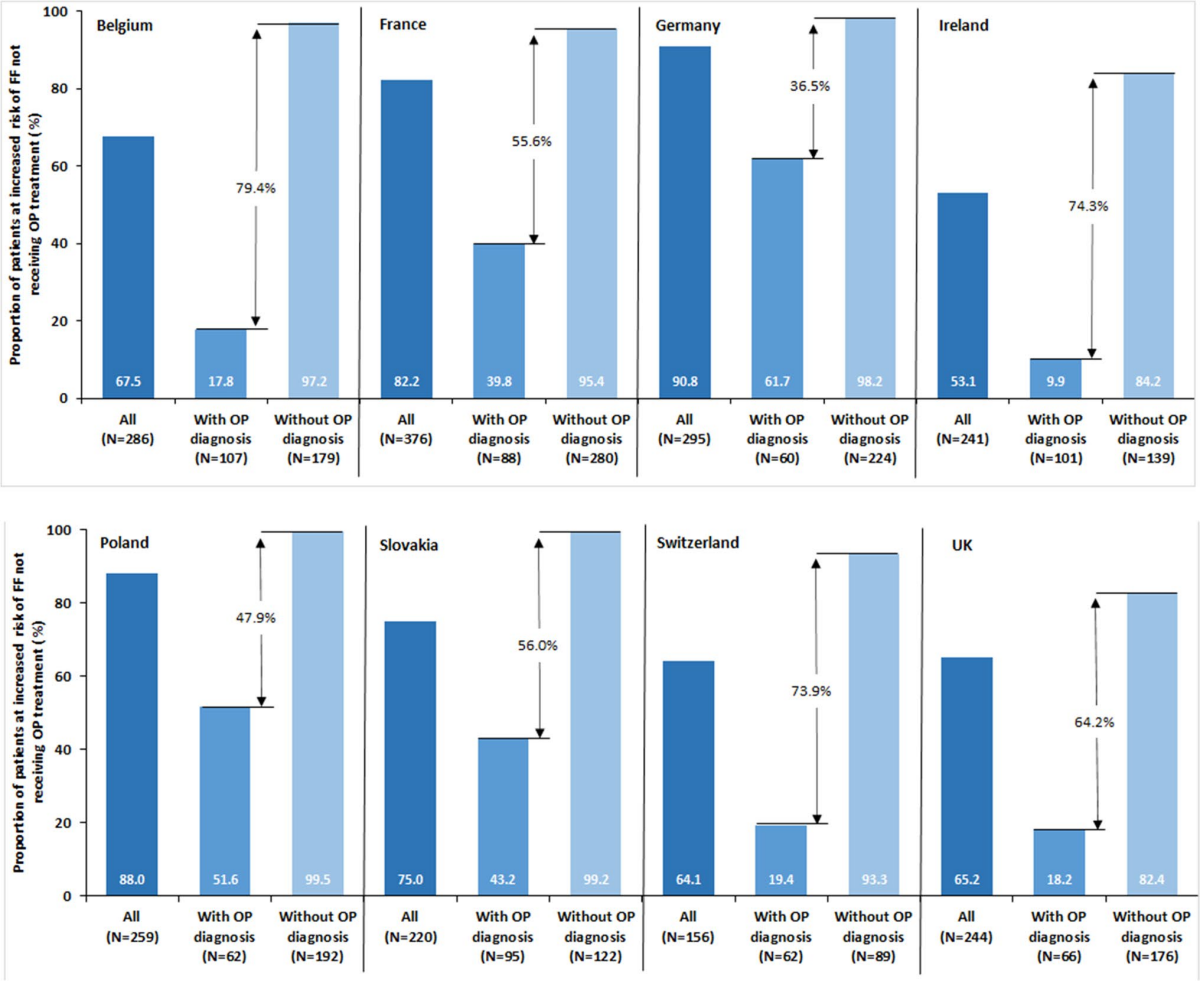
Osteoporosis treatment gap in elderly women in 8 European countries by osteoporosis diagnosis. For each country, the bars show the proportion (%) of patients at increased risk of fragility fracture who were not receiving treatment for osteoporosis (i.e., the treatment gap). Numbers within columns represent the treatment gap. Number (*N*) at increased risk of fragility fracture is shown beneath each column. “All”: Treatment gap overall; “With OP Diagnosis”: Treatment gap in patients who had an osteoporosis diagnosis; “Without OP Diagnosis”: Treatment gap in patients who did not have an osteoporosis diagnosis. The percentage shown within arrows is the difference in treatment gap between those with an OP diagnosis and those without. *FF,* fragility fracture; *OP,* osteoporosis

The primary outcome (OP treatment gap in patients at increased risk of FF) varied across countries (Fig. 1 and Table 2). Germany had the highest treatment gap (90.8% [95% CI: 87.0–93.9%], while Ireland had the lowest (53.1% [46.6–59.5%]). The treatment gap was notably reduced in patients who had an OP diagnosis (Fig. 1; arrows display difference in treatment gap between those with an OP diagnosis and those without). The difference ranged from 36.5% in Germany to 79.4% in Belgium.

**Table 2:** Primary outcome (treatment gap) for patients with history of fracture, *T*-score ≤  − 2.5, or who exceeded country-specific FRAX threshold

Increased risk of fragility fracture	Belgium	France	Germany	Ireland	Poland	Slovakia	Switzerland	UK	Total
**All patients**									
*N*	286	376	295	241	259	220	156	244	2077
*n* (%)	193 (67.5)	309 (82.2)	268 (90.8)	128 (53.1)	228 (88.0)	165 (75.0)	100 (64.1)	159 (65.2)	1550 (74.6)
(95% CI)	(61.7, 72.9)	(77.9, 85.9)	(87.0, 93.9)	(46.6, 59.6)	(83.4, 91.7)	(68.7, 80.6)	(56.0, 71.6)	(58.8, 71.1)	(72.7, 76.5)
**Any fracture**									
*N*	159	148	151	155	156	178	96	157	1200
*n* (%)	102 (64.2)	112 (75.7)	129 (85.4)	87 (56.1)	134 (85.9)	138 (77.5)	55 (57.3)	100 (63.7)	857 (71.4)
(95% CI)	(56.17, 71.59)	(67.95, 82.35)	(78.78, 90.64)	(47.94, 64.08)	(79.43, 90.95)	(70.68, 83.43)	(46.78, 67.34)	(55.65, 71.21)	(68.77, 73.96)
**Hip fracture**									
*N*	22	20	17	21	16	19	8	22	145
*n* (%)	10 (45.5)	12 (60.0)	16 (94.1)	9 (42.9)	12 (75.0)	15 (78.9)	7 (87.5)	12 (54.5)	93 (64.1)
(95% CI)	(24.39, 67.79)	(36.05, 80.88)	(71.31, 99.85)	(21.82, 65.98)	(47.62, 92.73)	(54.43, 93.95)	(47.35, 99.68)	(32.21, 75.61)	(55.76, 71.93)
**Spine fracture**									
*N*	26	21	28	14	22	24	31	12	178
*n* (%)	4 (15.4)	13 (61.9)	18 (64.3)	4 (28.6)	12 (54.5)	13 (54.2)	9 (29.0)	2 (16.7)	75 (42.1)
(95% CI)	(4.36, 34.87)	(38.44, 81.89)	(44.07, 81.36)	(8.39, 58.10)	(32.21, 75.61)	(32.82, 74.45)	(14.22, 48.04)	(2.09, 48.41)	(34.79, 49.75)
***T*-score ≤ -2.5**									
*N*	64	19	17	62	20	44	54	38	318
*n* (%)	10 (15.6)	4 (21.1)	9 (52.9)	5 (8.1)	4 (20.0)	15 (34.1)	13 (24.1)	6 (15.8)	66 (20.8)
(95% CI)	(7.76, 26.86)	(6.05, 45.57)	(27.81, 77.02)	(2.67, 17.83)	(5.73, 43.66)	(20.49, 49.92)	(13.49, 37.64)	(6.02, 31.25)	(16.43, 25.63)
**Exceeded FRAX threshold**									
*N*	255	370	275	195	219	149	140	211	1814
*n* (%)	178 (69.8)	305 (82.4)	252 (91.6)	108 (55.4)	197 (90.0)	114 (76.5)	86 (61.4)	137 (64.9)	1377 (75.9)
(95% CI)	(63.77, 75.38)	(78.16, 86.17)	(87.71, 94.62)	(48.11, 62.49)	(85.19, 93.60)	(68.88, 83.06)	(52.84, 69.53)	(58.08, 71.35)	(73.87, 77.86)

*BMD*, bone mineral density; *CI*, confidence interval; *DXA*, dual-energy X-ray absorptiometry; *FF*, fragility fracture; *FRAX*, Fracture Risk Assessment Tool; *OP*, osteoporosis

*N*, number of patients who are at increased risk of FF—using definition 1 (without BMD). *n*, number of patients not receiving any OP medication. Percentages based on number of patients enrolled who are at increased risk of FF

^a^A patient will be considered to be at increased risk of FF if ≥ 1 of the 3 following criteria are met: (1) had a history of fracture; (2) 10-year probability of hip fracture without BMD > country-specific threshold and 10-year probability of major osteoporotic fracture without BMD > country-specific threshold; (3) BMD *T*-score ≤  − 2.5 for any of lumbar spine/total hip/femoral neck

Across countries, the treatment gap was lower in those with spine fracture than for all patients at increased risk of FF (Table 2), with the lowest treatment gap in this group observed in Belgium and the UK (15–17%). The effect of hip fracture was less pronounced: the treatment gap was lower in those with hip fracture than all patients in Belgium, France, Ireland, Poland, and the UK; but higher in Germany, Slovakia, and Switzerland.

In all countries, the treatment gap was substantially lower in those with *T*-scores ≤ -2.5 than all patients at increased risk of FF (Table 2). Across countries, the treatment gap in patients who exceeded the country-specific FRAX threshold was similar to the treatment gap for all patients at increased risk of FF.

### OP medication use

For all countries except Switzerland, oral BPs were the most commonly used type of OP medication (ranging from 32.1% [of those using any OP medication] in Switzerland to 96.1% in the UK; Table 3). Belgium, Germany, Ireland, and Switzerland had relatively high proportions of patients using denosumab (39.9 to 55.4% of those using any OP medication) while France, Poland, and the UK had comparatively low proportions (1.0 to 17.9%).

**Table 3:** OP medication use

Any OP medication, *n* (%)	Belgium (*N* = 110)	France (*N* = 75)	Germany (*N* = 32)	Ireland (*N* = 143)	Poland (*N* = 39)	Slovakia (*N* = 69)	Switzerland (*N* = 56)	UK (*N* = 102)	Total (*N* = 626)
Denosumab	46 (41.8)	12 (16.0)	15 (46.9)	57 (39.9)	7 (17.9)	18 (26.1)	31 (55.4)	1 (1.0)	187 (29.9)
Oral BP	65 (59.1)	44 (58.7)	19 (59.4)	106 (74.1)	34 (87.2)	38 (55.1)	18 (32.1)	98 (96.1)	422 (67.4)
Parenteral BP	24 (21.8)	7 (9.3)	5 (15.6)	3 (2.1)	3 (7.7)	18 (26.1)	19 (33.9)	6 (5.9)	85 (13.6)
PTH	2 (1.8)	0 (0.0)	0 (0.0)	4 (2.8)	0 (0.0)	0 (0.0)	2 (3.6)	0 (0.0)	8 (1.3)
SERM	14 (12.7)	14 (18.7)	1 (3.1)	4 (2.8)	0 (0.0)	5 (7.2)	1 (1.8)	2 (2.0)	41 (6.5)
Strontium	2 (1.8)	12 (16.0)	2 (6.3)	12 (8.4)	0 (0.0)	6 (8.7)	0 (0.0)	6 (5.9)	40 (6.4)

*BPs*, bisphosphonates; *OP*, osteoporosis; *PTH*, parathyroid hormone; *RANKL*, receptor activator of nuclear factor kappa-Β ligand; *SERMs*, selective estrogen receptor modulators

*N*, number of patients enrolled who are receiving or have received any OP medication in the last 10 years

**Note:** Percentages add up to more than 100% because patients may be counted under more than one medication; and both concurrent and previous medications are included. OP medication use is derived considering SERMs, oral BPs, parenteral BPs, strontium, PTH, and anti-RANKL/denosumab medications

## Discussion

Our study found a large treatment gap for women aged ≥ 70 years at increased risk of FF who were in routine primary care across 8 countries in Europe. While the treatment gap varied (53.1% in Ireland to 90.8% in Germany), it was above 50% in all 8 countries. Across countries, the treatment gap was lower in patients with an OP diagnosis, with the difference between patients with and without a diagnosis ranging from 36.5% in Germany to 79.4% in Belgium.

Studies in other regions of the world have also identified a large OP treatment gap among older or postmenopausal women (in these studies, the treatment gap was usually defined as the proportion who did not receive OP medication in a defined period after a primary fracture or an OP diagnosis). In the USA, the treatment gap was reported as 72.1% (data from the Women’s Health Initiative study [15]) and 81. 4% [16]; in Asia, treatment gap varied from 64.5% in a multi-country study in China and South-East Asia [17] to as high as 98.6% in a cross-sectional study in China [18].

The demographic characteristics of the European women over ≥ 70 recruited in this study were comparable across the 8 countries. The proportion of patients with an OP diagnosis varied from 15.0% (Poland) to 30.2% (Switzerland). Across countries, less than a third of the patients had had a DXA scan, except in Belgium, Switzerland, and Ireland (37.2 to 44.6%). The proportion of patients at increased risk of FF varied from 41.2% (Slovakia) to 76.1% (Switzerland). Of this subset of patients, < 50% had a diagnosis of OP (from 20.3% in Germany to 43.2% in Slovakia). Previous fracture was the most commonly reported clinical risk factor for FF, with similar prevalence across all countries (27.3 to 33.3%) except Switzerland (46.8%). Other risk factors, such as parental hip fracture, rheumatoid arthritis, glucocorticoid use, smoking, alcohol intake, and median *T*-score, varied widely. Ten-year probability of hip and major OP fracture was similar in all countries (6.1 to 8.8% for hip fracture and 14.5 to 18.3% for major OP fracture), except for Poland (3.7% for hip fracture and 9.5% of major OP fracture) and Switzerland (12.2% for hip fracture and 29.3% for major OP). Overall, the rank order of the prevalence of risk factors was similar in all countries though absolute prevalence showed some variation. Despite this variability, the absolute FRAX probabilities were fairly similar, with the possible exception of Poland and Switzerland, showing the predominant impact of prior fracture.

An earlier study [10], which used a different methodology and was not limited to primary care, reported lower treatment gaps for the countries in our study; however, that study also identified Ireland as having the lowest treatment gap (26%). The treatment gap in our study was substantially lower in patients diagnosed with OP; however, the gap in these patients also varied across countries, from 9.9% in Ireland to 61.7% in Germany. This suggests there are substantial differences across Europe in how well women eligible for OP treatment are identified, as well as differences in treatment uptake in those who are diagnosed with OP. In some countries, such as Germany, most OP patients may be treated by orthopedic physicians instead of general practitioners (GPs), which could contribute to a low rate of DXA measurements and a high treatment gap. The involvement of GPs is an important aspect of OP prevention and management. Two studies in France revealed that physician’s attitudes and lack of awareness of OP can be a significant barrier to identifying and treating patients at risk for fracture [19, 20]. Physicians’ attitudes also influence those of the OP patients themselves, which can have an impact on treatment adherence [19]. A qualitative study in Sweden [21] found that primary care physicians perceive OP as a silent disease that is overshadowed by other conditions and seen as a low priority. The recently published SCOPE scorecard provides a detailed comparison of OP epidemiology and care across Europe, including estimate of the treatment gap in those aged 50 or more [22]. The treatment gap in Europe was estimated to be 71% overall, and as for our study was lowest in Ireland (32%). Gaps in knowledge, diverse opinions on who should be responsible for management, and uncertainty about treatment protocol could affect the management of OP. Furthermore, FRAX use may be limited by absence of linkage to electronic medical records.

The FRAX uptake reported in SCOPE was substantially lower in those countries where we found a higher treatment gap in our study (Germany, France, Poland, and Slovakia) [22]. In our study, the three countries with the lowest rates of DXA scans (Poland [8.3%], France [11.4%], and Germany [12.3%]) also had the highest treatment gap overall (88.0%, 82.2%, and 90.8%, respectively); conversely, Ireland, with the highest rate of scans (44.6%), had the lowest treatment gap (53.1%). Availability of DXA is similar across all countries except Poland and the UK, and scans are fully or partially reimbursed in all the countries [22]; therefore, differences observed in rates of DXA between countries may reflect differences in awareness of OP and fracture risk, which in turn drives the treatment gap.

The impact of clinical risk factors on the treatment gap varied between countries. The countries with highest treatment gap overall (Poland and Germany) also had high treatment gaps in patients who exceeded the country-specific FRAX threshold (90.0% and 91.6%), and in patients with hip fractures (75.0% and 94.1%); conversely, Ireland (with the lowest treatment gap overall) had the lowest treatment gap in patients who exceeded the FRAX threshold as well as in patients with hip fracture (55.4% and 42.9%). Ireland also had the lowest treatment gap in patients with *T*-scores less than − 2.5 (8.1%). These data suggest that although physicians consider known clinical risk factors when making treatment decisions, there is variability in the weighting given to individual risk factors when identifying patients for treatment in some countries.

Among OP medications, oral BPs were used at high rates in most countries in our study (32.1 to 96.1% of those using any OP medication), reflecting their use as a standard first-line treatment. Denosumab was used at relatively high rates in Belgium, Germany, Ireland, and Switzerland (39.9 to 55.4%), and at low rates in other countries (1.0 to 26.1%), which may be due to differences in access or reimbursement. In general, the other medications assessed (parenteral BP, PTH, SERM, and strontium) were used at low rates. Treatment patterns reported in the literature are consistent with these findings. BPs were reported as by far the dominant treatment in UK primary care for the period 2010 to 2015 [23]. An analysis of German pharmacy prescription data showed that most first-time prescriptions between 2010 and 2014 were for oral BPs, but denosumab treatment was more common than found in the UK study [24].

This study has some important strengths as well as limitations. GPs willing and able to participate in observational studies could represent a specialized subgroup; therefore, as noted earlier [13], steps were taken to reduce bias during recruitment. The prevalence of risk factors and comorbidities agreed with that reported earlier [25–30], indicating that the study population is typical for this demographic. However, since the patients were women ≥ 70 years visiting their GP, they may not be representative of the general population. The study was not also designed to establish causality between the prevalence of risk factors and treatment gap. All analyses were descriptive; no statistical comparisons were made across countries. The sample sizes were small in relation to the total number of women with OP in the countries in the study, and therefore the results may not be representative. This is especially true for Switzerland, which had the smallest number of subjects (205, across 6 sites).

In conclusion, our study highlights differences across countries in Europe with respect to clinical risk factors for fracture, DXA scanning, and the rates of OP diagnosis. The OP treatment gap differed but still fewer than 50% of women at increased risk of FF were treated in any country. Our findings suggest that there is variable success across Europe in identifying and treating women at risk for fracture. More emphasis needs to be placed on BMD measurements as a tool for screening elderly women, and on the use of FRAX or other risk assessment tools. It is also important that patients and physicians across Europe be educated about the importance of screening and prevention measures for OP. Finally, further studies are needed to understand the reasons for large treatment gaps in primary care, to identify potential solutions and appropriate steps to improve the treatment of OP and lower the occurrence of fracture in elderly women.

## Supplementary Information

Below is the link to the electronic supplementary material.

Supplementary file1 (DOCX 24 KB)
