# Supplementary material for: Prevalence of FRAX risk factors and the osteoporosis treatment gap among women ≥ 70 years of age in routine primary care across 8 countries in Europe
Source: Arch Osteoporos. 2022 Jan 22;17(1):20. doi: 10.1007/s11657-021-01048-8 (PMC8783912; doi:10.1007/s11657-021-01048-8)
Supplement: Supplementary file 1 — Supplementary file1 (DOCX 24 KB) [file 11657_2021_1048_MOESM1_ESM.docx]

**Prevalence of FRAX risk factors and the osteoporosis treatment gap among women ≥70 years of age** **in routine primary care across 8 countries in Europe**

Authors: Eugene McCloskey, Jeetandera Rathi, Stephane Heijmans, Mark Blagden, Bernard Cortet, Edward Czerwinski, Peyman Hadji, Juraj Payer, Kerry Palmer, Robert Stad, James O’Kelly, Socrates Papapoulos

**Corresponding Author: Eugene McCloskey**

*Address:* Centre for Metabolic Bone Diseases, University of Sheffield, Sheffield, UK

Phone: +44 (0)114 271 4705, Email: [E.V.McCloskey@sheffield.ac.uk](mailto:E.V.McCloskey@sheffield.ac.uk)

## ***Electronic Supplementary Information***

This file contains tables displaying FRAX probability thresholds for treatment for each country in the study (Table S1), and baseline characteristics of patients with increased risk of fragility fracture, by country (Table S2).

Table S1: Country-specific 10-Year Probability Thresholds Calculated by FRAX

| **Country** | **Threshold (%)** | |
| --- | --- | --- |
|  | **10-year probability of hip fracture without BMD** | **10-year probability of major osteoporotic fracture without BMD** |
| Belgium | 5 | 18 |
| France | 4.3 | 13 |
| Germany | 5.3 | 16 |
| Ireland | 6.5 | 20 |
| Netherlands | 4 | 13 |
| Poland | 3.3 | 10 |
| Slovakia | 6.7 | 19 |
| Sweden | 9 | 25 |
| Switzerland | 6.1 | 25 |
| UK | 5 | 20 |

BMD = bone mineral density; FRAX = Fracture Risk Assessment Tool.

Table S2: Baseline characteristics of patients with increased risk of FF

| **Increased Risk of Fragility Fracture^a^** | **Belgium (N = 286)** | **France (N = 376)** | **Germany (N = 295)** | **Ireland (N = 241)** | **Poland (N = 259)** | **Slovakia (N = 220)** | **Switzerland (N = 156)** | **UK (N = 244)** | **Total (N = 2077)** |
| --- | --- | --- | --- | --- | --- | --- | --- | --- | --- |
| Age, median (Q1, Q3), years | 81.0  75.0, 85.0 | 82.0  78.0, 86.0 | 80.0  77.0, 83.0 | 78.0  74.0, 82.0 | 79.0  75.0, 84.0 | 77.0  74.0, 81.0 | 79.0  74.0, 84.0 | 80.0  74.5, 85.0 | 80.0  75.0, 84.0 |
| BMI, median (Q1, Q3), kg/m^2^ | 25.9  23.0, 29.0 | 24.9  22.2, 28.0 | 25.1  22.5, 28.0 | 25.6  22.5, 29.7 | 27.0  24.0, 30.3**^*^** | 27.9  24.7, 31.6 | 24.6  21.5, 28.0 | 25.1  22.6, 28.8 | 25.7  22.9, 29.3**^**^** |
| Reason for consultation - n (%) |  |  |  |  |  |  |  |  |  |
| Follow-up to known disease | 134 (46.9) | 125 (33.2) | 210 (71.2) | 103 (42.7) | 92 (35.5) | 147 (66.8) | 108 (69.2) | 142 (58.2) | 1061 (51.1) |
| Medication refill | 86 (30.1) | 183 (48.7) | 34 (11.5) | 20 (8.3) | 82 (31.7) | 35 (15.9) | 17 (10.9) | 11 (4.5) | 468 (22.5) |
| New symptoms/complaints | 57 (19.9) | 48 (12.8) | 47 (15.9) | 77 (32.0) | 75 (29.0) | 29 (13.2) | 29 (18.6) | 80 (32.8) | 442 (21.3) |
| Other | 9 (3.1) | 20 (5.3) | 4 (1.4) | 41 (17.0) | 10 (3.9) | 9 (4.1) | 2 (1.3) | 11 (4.5) | 106 (5.1) |
| At least one comorbidity, n (%) | 249 (87.1) | 335 (89.1) | 260 (88.1) | 196 (81.3) | 249 (96.1) | 209 (95.0) | 141 (90.4) | 215 (88.1) | 1854 (89.3) |
| RA | 15 (5.2) | 23 (6.1) | 24 (8.1) | 14 (5.8) | 15 (5.8) | 16 (7.3) | 6 (3.8) | 17 (7.0) | 130 (6.3) |
| Diabetes | 51 (17.8) | 60 (16.0) | 98 (33.2) | 30 (12.4) | 75 (29.0) | 72 (32.7) | 31 (19.9) | 35 (14.3) | 452 (21.8) |
| Hypertension | 208 (72.7) | 257 (68.4) | 240 (81.4) | 154 (63.9) | 226 (87.3) | 203 (92.3) | 103 (66.0) | 148 (60.7) | 1539 (74.1) |
| Osteoarthritis | 147 (51.4) | 254 (67.6) | 71 (24.1) | 117 (48.5) | 124 (47.9) | 59 (26.8) | 99 (63.5) | 136 (55.7) | 1007 (48.5) |
| COPD | 23 (8.0) | 26 (6.9) | 32 (10.8) | 23 (9.5) | 21 (8.1) | 16 (7.3) | 13 (8.3) | 33 (13.5) | 187 (9.0) |
| Clinical risk factors for FF – n (%) |  |  |  |  |  |  |  |  |  |
| Previous fracture | 159 (55.6) | 148 (39.4) | 151 (51.2) | 155 (64.3) | 156 (60.2) | 178 (80.9) | 96 (61.5) | 157 (64.3) | 1200 (57.8) |
| Parental hip fracture | 44 (15.4) | 65 (17.3) | 38 (12.9) | 29 (12.0) | 33 (12.7) | 30 (13.6) | 27 (17.3) | 36 (14.8) | 302 (14.5) |
| Current smoker | 21 (7.3) | 18 (4.8) | 21 (7.1) | 18 (7.5) | 15 (5.8) | 10 (4.5) | 13 (8.3) | 16 (6.6) | 132 (6.4) |
| Glucocorticoid use | 18 (6.3) | 17 (4.5) | 21 (7.1) | 31 (12.9) | 18 (6.9) | 7 (3.2) | 14 (9.0) | 31 (12.7) | 157 (7.6) |
| Rheumatoid arthritis | 15 (5.2) | 23 (6.1) | 24 (8.1) | 14 (5.8) | 15 (5.8) | 16 (7.3) | 6 (3.8) | 17 (7.0) | 130 (6.3) |
| Alcohol (≥3 units per day) | 4 (1.4) | 3 (0.8) | 2 (0.7) | 5 (2.1) | 0 (0.0) | 2 (0.9) | 6 (3.8) | 10 (4.1) | 32 (1.5) |
| Femoral neck T-score – median (Q1, Q3) [n] | -2.1 (-2.6, -1.5) [85] | -1.7 (-2.3, -0.6) [28] | -1.3 (-2.4, -0.6) [37] | -1.8 (-2.4, -1.1) [136] | -2.5 (-3.0, -1.9) [14] | -1.7 (-2.3, -1.1) [75] | -2.2 (-2.6, -1.4) [79] | -1.4 (-2.3, -0.9) [64] | -1.8 (-2.5, -1.1) [518] |
| 10-year fracture probability without BMD – median (Q1, Q3), % |  |  |  |  |  |  |  |  |  |
| Hip fracture | 12.9 (9.7, 17.4) | 11.6 (8.3, 17.5) | 11.0 (8.5, 14.6) | 12.7 (8.5, 17.0) | 6.4 (4.5, 9.3) **^*^** | 10.0 (6.7, 13.1) | 14.5 (9.9, 24.1) | 12.2 (8.4, 17.0) | 11.2 (7.6, 16.2) **^**^** |
| Major OP fracture | 23.0 (20.0, 30.2) | 23.1 (17.6, 30.0) | 21.6 (17.7, 27.6) | 25.2 (20.8, 31.2) | 13.7 (11.4, 17.6) **^*^** | 22.1 (17.7, 25.8) | 32.8 (27.7, 40.9) | 24.9 (21.1, 31.8) | 22.8 (18.0, 29.6) **^**^** |

BMD = bone mineral density; BMI = body mass index; COPD = Chronic obstructive pulmonary disease; FF = fragility fracture; OP = osteoporosis; RA = Rheumatoid arthritis.
N = Number of patients enrolled in full analysis set. Percentages based on total number of patients in respective increased risk of FF category as per definition 1.

^*^n = 253. ^**^n = 2071.

^a^ A patient will be considered to be at increased risk of FF if ≥1 of the 3 following criteria are met: (1) had a history of fracture; (2) 10-year probability of hip fracture without BMD > country-specific threshold and 10-year probability of major osteoporotic fracture without BMD > country-specific threshold; (3) BMD T-score ≤-2.5 for any of lumbar spine/total hip/femoral neck.
